# Scopus database: a review

**DOI:** 10.1186/1742-5581-3-1

**Published:** 2006-03-08

**Authors:** Judy F Burnham

**Affiliations:** 1 University of South Alabama Biomedical Library, 316 BLB, Mobile, AL 36688, USA

## Abstract

The Scopus database provides access to STM journal articles and the references included in those articles, allowing the searcher to search both forward and backward in time. The database can be used for collection development as well as for research. This review provides information on the key points of the database and compares it to Web of Science. Neither database is inclusive, but complements each other. If a library can only afford one, choice must be based in institutional needs.

## 

Scopus is an abstract and indexing database with full-text links that is produced by the Elsevier Co. The name, Scopus, was inspired by the bird, Hammerkop (*Scopus umbretta*), which reportedly has excellent navigation skills. The database, in development for two years, was developed working with 21 research institutions and more than 300 researchers and librarians. The verbal and behavioral feedback of these librarians and researches was analyzed and used to improve the product.

## Content of Scopus

Scopus developers claim to index over 14,000 STM and social science titles from 4000 publishers, stating that it is the "largest single abstract and indexing database ever built". The database claims 4600 health science titles are indexed including 100% MEDLINE coverage, 100% of EMBASE coverage and 100% of Compendex coverage. The list of titles indexed is selected based on user demand and market research. It contains 27 million abstracts with citations back to 1966. In addition to American journals, it includes European and Asia Pacific literature in both English and non-English. Indexing includes CAS registry numbers, MeSH terms, EMTREE terms and supplemental key terms added by indexers.

Some features of Scopus include:

• Links to both citing and cited documents, allowing the user to go both forwards and backwards in time.

• Open access titles are included in the index

• Indexes web pages and patents, with a claim to over 167 million relevant web pages.

• OpenURL compliant and works with any link resolver, using image-based linking.

• Runs an entitlement check prior to returning a full-text image if the article if available to the user.

• Can link to the publisher's web site to view the document.

• Developers claim that "citation accuracy is achieved by using state-of-the-art technology, with 99% of citing references and citing articles matched exactly."

• For statistics on usage, Scopus delivers customer-specific usage reports which will be COUNTER compliant. (COUNTER [**C**ounting **O**nline **U**sage of **N**etworked **E**lectronic **R**esources] is an international standard for the recording and exchange of online usage statistics.)

• Offers both on- and off-site training as well as web-based training and online tutorials. The database website offers quick reference guides, tips, etc. (in English and other languages) and offers online technical support.

• Works equally well with Internet Explorer, Netscape and Foxfire.

## Search of Scopus

There are two search modes for Scopus – Basic and Advanced

• The Basic Search uses fill-in and drop-down boxes to search various fields.

◦ The search can be limited to date, document type, subject area or recent updates. Once the citations are retrieved, results can be excluded according to set criteria.

◦ A particular author can be searched by name. After searching, the user will get a list of possible matches. One or more names can be selected from this list. The user can search for variants of a name and for parts of a name (i.e., a hyphenated name.)

• With the Advanced Search, the user can use Boolean operators and nesting using field labels.

Other features of Scopus include:

• The list of journals can be browsed by title or by subject area for journals published since 1996.

• Results from searches can be sorted by date, relevance, author(s), source, title and by "Cited By". The "Cited By" sort will list the documents according to the number of citations received. After applying limits or exclusions, the list can be re-sorted.

• Search can be conducted within results. The Search History allows the user to view searches that have been conducted, combine searches or save searches.

• All or a selection of the citations can be printed. They can also be exported into bibliographic management software or e-mailed.

• The user can add selected documents to a created list which can then be exported, printed, etc.

• Retrieval can be limited to Scopus citations only, web citations only, patent citations, or to Scopus and web combined. If limited to Scopus citations, the retrieval will include the abstract, full text (if available), references, and citations to article being examined. When limited to web citations, the database uses Scirus, a science-only Internet search engine for retrieval.

• Each article will include a link to library entitled full-text so it can be retrieved from the results list, the abstract or the references. An icon appears if the user as rights to the full-text.

• The "Cited By" lists articles that cite the article being examined.

• The "Related" journals offer a list of papers that share the same references with the paper being examined.

• In addition, the product offers an alert service, both for new articles being added on a particular subject and for new articles being added that cite a particular article

• "My Profile" allows the user to save searches to run on a future date.

## Scopus vs Web of Science*

Table 1 shows a comparison of coverage between Scopus and Web of Science.

**Table 1:** Comparison of Scopus vs Web of Science

	**Web of Science (SCI)**	**Scopus**
**Coverage**	1992-date	1966-date (abstracts)
	1900-date	1996-date (cited references)
**# of Journals Indexed**	8700	14,000
**# of Citations**	36.1 million	27 million
**Update Schedule**	Weekly	Updated daily
**Subjects covered**	Life sciences, clinical medicine, animal & plant biology, biotechnology, agriculture, environmental sciences, physics, chemistry, earth sciences, mathematics, engineering, technology, computer science	5900 titles in life and health sciences; chemistry, physics, math, engineering, social sciences, psychology, economics, biological, agricultural, environmental, general sciences
**Geographic coverage**	80 countries	60% of titles are from countries other than USA
**Indexing**	Author defined keywords and KeyWordsPlus	EMTREE, MESH and others

### Ease of search

#### WOS

The opening screen allows the user to do a general search, a cited reference search or an advanced search. The general search has boxes for the user to fill in (topic, source title, etc.) and allows limits by language and document type. The Advance search allows the user to fill in a box using Boolean, again allowing the user to limit by language or document type. The Cited Reference Search allows the user to search by cited author, cited title or cited year. Examples are given to guide the user. These search options are available from all screens.

#### Scopus

Basic Search allows the user to enter term into search box and choose field to be searched from drop down box. Terms can be combined using Boolean. Limits can be set date range, document type and update frequency. Advanced search allows for open entry of search terms, with examples given. The Advanced Search box must be used when combining more than two terms.

### Author

#### WOS

In the General Search area, a box is available for author searching. Examples are given for correct format.

#### Scopus

A tab for "Author" allows for easy searching of the author with a separate box for first and last name and examples given.

### Cited reference

#### WOS

Cited Reference Search is one of the options for searching from the opening screen. The user can then choose from Cited Author, Cited Work or Cited Year.

#### Scopus

Cited authors, titles, etc. can be searched from the Advanced Search interface by using the appropriate codes, i.e., REFAUTH, REFTITLE, etc.

### Viewing records

#### WOS

Results are presented in citation format and can be sorted by date, relevancy, times cited, author or source title.

#### Scopus

Results sorted according to Scopus, Web, Patents and Combined tabs, with the default to the Scopus list. Results are presented in tabular form by Date, Document, Author(s), Source Title and Cited By. Any of the columns can be sorted, allowing for quick analysis of the results. A "Refine Results" area allows the user to filter results using either a "limit" or "exclude" tool for Source Title, Author, Year, Document Type or Subject Area. The top three in each category is listed along with a link to "more". This may seem like a lot of information on a screen, but there is enough white space so that the view is clear and not confusing.

### Linking to full text

#### WOS

Clicking on the "View Full Text" button links to EbscoHost where the user can link to the full-text if the library subscribes to that resource or the user can link to other sources, i.e., ScienceDirect for the full-text.

#### Scopus

Links from the citation are available for abstract, references and/or full-text. Author names are also linked to other articles by that author. A link resolver allows the user to link to the full-text. Links from the Cited By column are to articles that have cited the original article. When viewing the References, Cited By statistics and links are available.

### Related records

#### WOS

When viewing one of the retrieved records, by clicking on "Find Related Records", the results are presented in a relevancy ranking based on the number of shared references. The number of cited references and the number of shared references (with the original article) are listed. From this screen, the user can view the shared references for each citation. These results are in a table format, but the entire citation can be viewed for those citations in the WOS database.

#### Scopus

When viewing one of the retrieved records, a click on "related records" will retrieve documents that have references in common with the article being viewed. When this list is sorted by relevance, those articles that share the most references will be displayed first.

### Saving, sorting, printing, etc

#### WOS

Once citations have been marked, they can be printed, saved, exported, e-mailed or ordered.

#### Scopus

Searches can be saved in an area called "My Profile" or can be saved as an alert, to be e-mailed to the user. From the Results page, the user can print, export, e-mail, or add citations to a list by first clicking on the box to the left of the citation and then clicking on the appropriate button.

**Figure 1:**
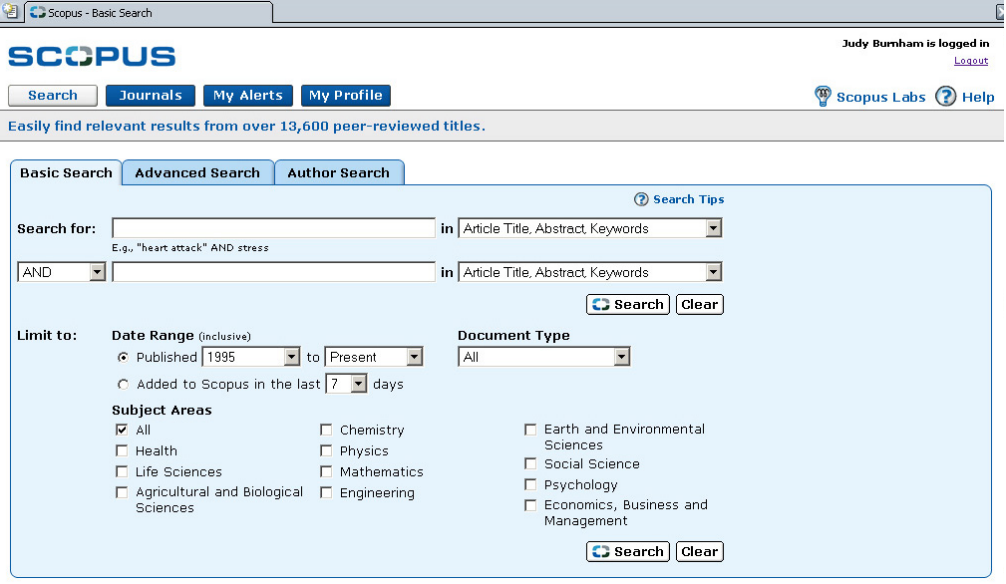
shows the Basic Search.

**Figure 2:**
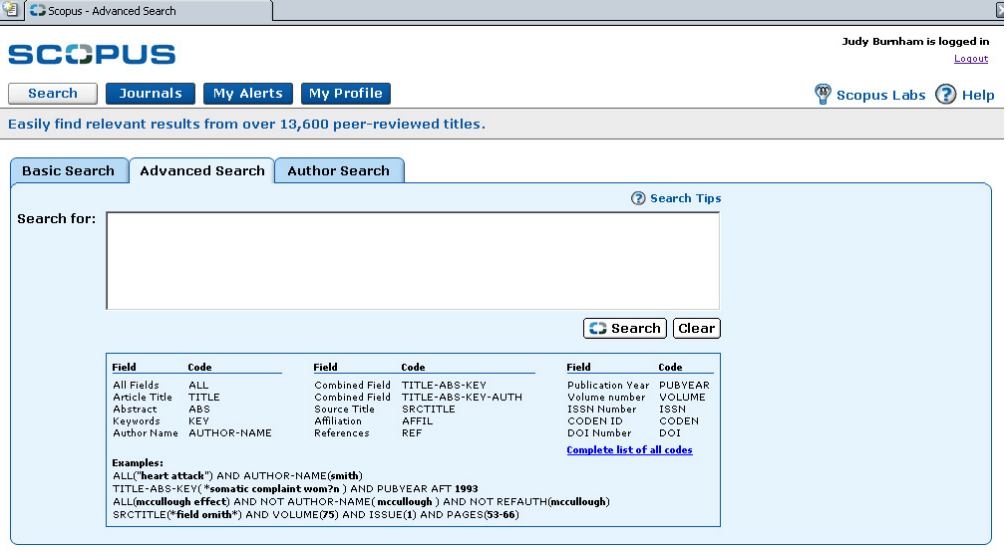
shows the Advanced Search format.

**Figure 3:**
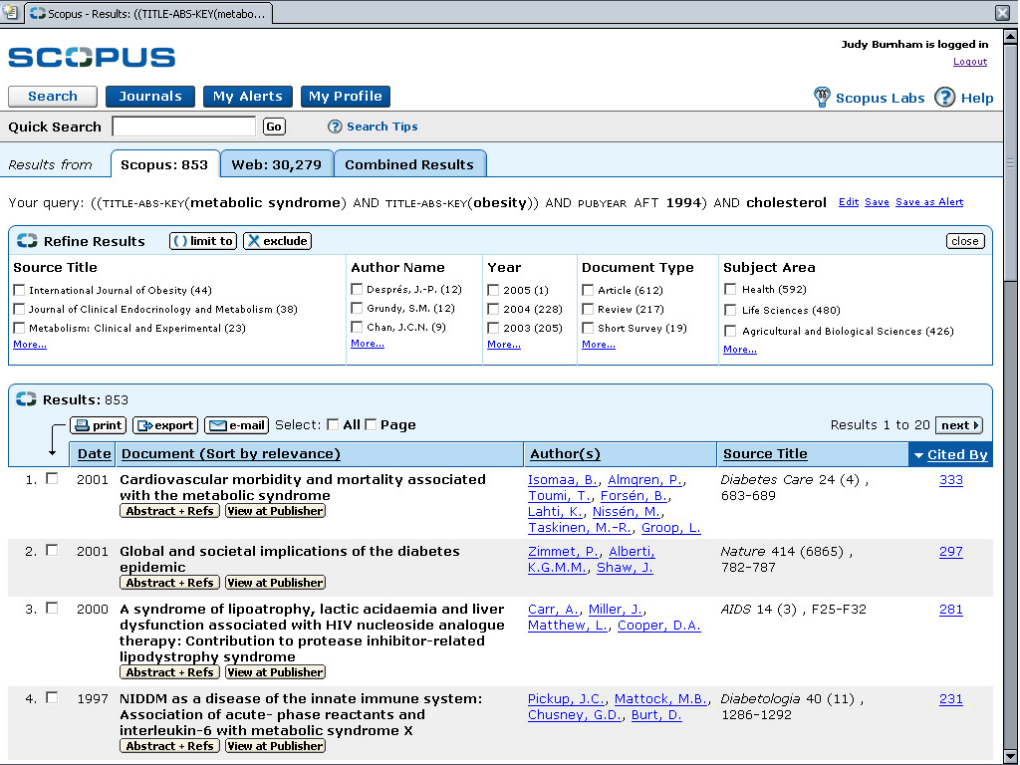
shows the Results list.

**Figure 4:**
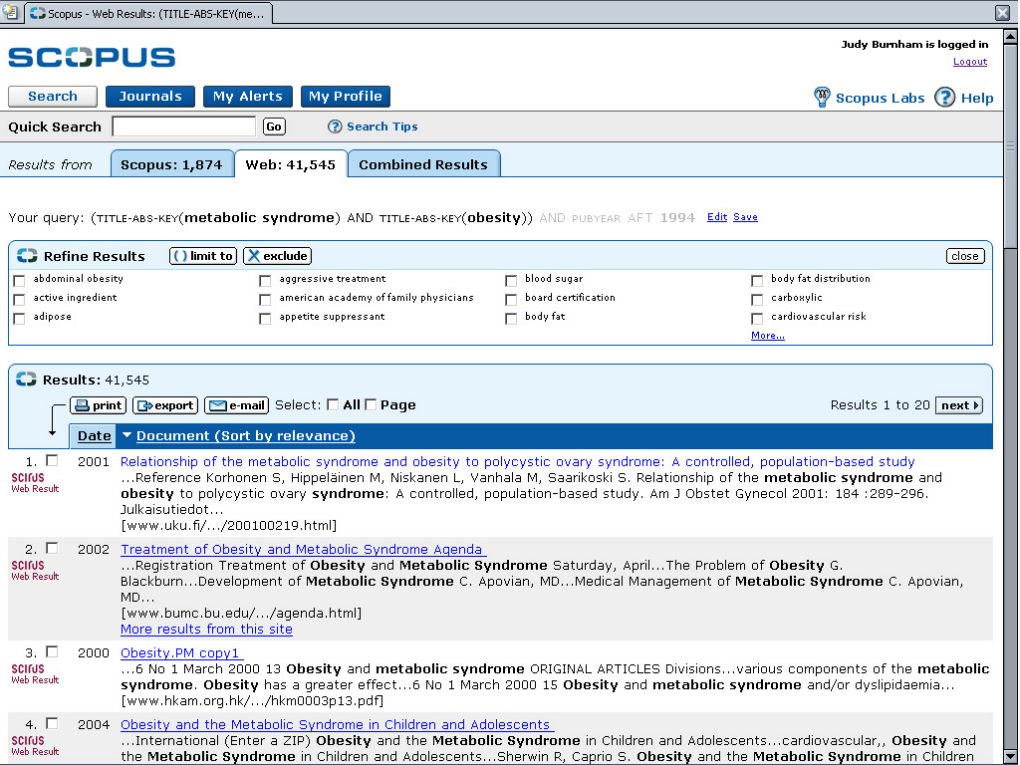
shows the Web Results list.

**Figure 5:**
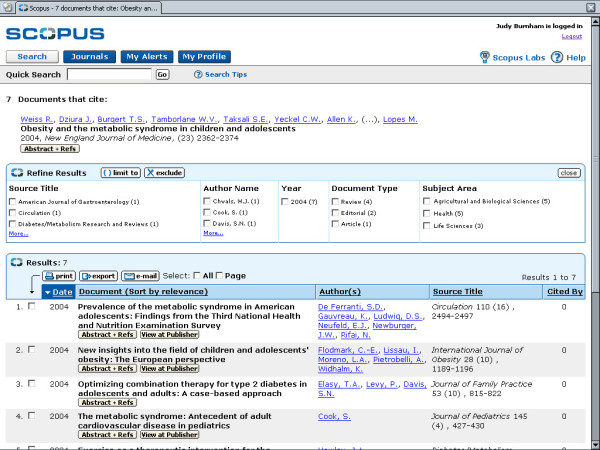
shows the Cited By Results.

### Analysis of citations

#### WOS

After a list of citations is retrieved, the user can click on the Analyze Records button to view rankings and histograms of the authors, journals, etc. for the set of records.

#### Scopus

The database currently only provides a cited-by count of records. However, they are working on a citation analysis tool that should be ready by the end of 2005.

### Help available

#### WOS

A tutorial is available on the Web of Knowledge and the Web of Science opening screens. Help buttons from each screen are specific to the screen being used, i.e., General Search, Search Results, etc. Also available is Help according to Contents and an A to Z Index.

#### Scopu

A "Help" button at the top of each screen leads the user to help topics that can be accesses by "Contents" and alphabetical index or by a search box. All help reviewed provided the answer desired.

## Comparison of searches between resources

Table 2 shows a comparison of searches between Scopus and Web of Science. In searching for combined subjects, for rare health conditions for known authors or for known journal titles, results are mixed. However, Scopus gave the best results overall for the sample searches.

**Table 2:** Comparison of searches between resources

**Title of Search**	# Ref Web of Science*	# Ref Scopus*
Arthritis and environment, 1992-date	405	395
Tubercular meningitis, 1992-date	21	76
Barik S*, 1992-date	93	126
American Journal of Cardiology, 1992-date	15,619	21,993

## Other uses of Scopus

From a collection development point-of-view, Scopus can be used to determine which journal is cited the most for a particular subject area. The company plans future development that will include a citation overview tool, with which the user can search a journal by year and get the total number of citations for that journal. With the demise of the Brandon-Hill list and if the library doesn't have access to the Journal Citation Reports from ISI, the database could be used to determine a list of core journals in a discipline.

Scopus is sold as an annual subscription and is based on FTE. They have customized pricing for very small and very large institutions.

## Conclusion

Scopus is easy to navigate, even for the novice user. If the user is familiar with search devices such as drop-down boxes and check boxes, searching will be a simple task with the intuitive search system. The ability to search both forward and backward from a particular citation would be very helpful to the researcher. The multidisciplinary aspect allows the researcher to easily search outside of his discipline.

One advantage of WOS over Scopus is the depth of coverage, with the full WOS database going back to 1945 and Scopus going back to 1966. However, Scopus and WOS compliment each others as neither resource is all inclusive. Libraries who can afford to will want to subscribe to both tools. Those who must choose must do so based on the needs of their individual library.

## Note

* Analysis of Scopus is based on c2005 trial edition. Analysis of Web of Science is based on Science Citation Index Expanded (SCI-EXPANDED)-1992-present

## Competing interests

The author(s) declare that they have no competing interests.

